# Editorial: Cultured Meat—Are We Getting it Right?

**DOI:** 10.3389/fnut.2021.675797

**Published:** 2021-06-14

**Authors:** Johannes le Coutre

**Affiliations:** School of Chemical Engineering, University of New South Wales (UNSW), Kensington, NSW, Australia

**Keywords:** cultured meat, cellular agriculture, food system, nutrition, sustainability

Since the launch of this Research Topic in the second half of 2019, the world has changed. The Covid-19 pandemic puts on hold many societal activities, upending normalcy for the majority of human life. Interestingly, not only has the pandemic impacted the progress of this Research Topic but it underscores the importance, opportunities, and relevance of cultured meats in a post-pandemic era.

The pandemic brings to light the extraordinary frailty in our global healthcare and food systems. We are just learning of the inter-relationship between diet and disease etiology and progression, and that certain dietary patterns can be associated with either an increase or decrease in disease risk and progression. Sugar intake, and gut microbial diversity appear to be only a few of the putative Covid-19 related nutritional metrics. More related to the Research Topic, we also witnessed Covid-19 outbreaks among food handlers in meat production and packaging plants, leading to plant closures.

At the outset of this Research Topic, we had written an overview outlining that the “*dietary consumption of meat is a hallmark of most human cultures and civilizations*.” However, maybe it is time to redefine this statement and consider that the “*dietary consumption of meat has been a hallmark of most human cultures and civilizations, but the twenty-first century necessitates more strategic technologies and sustainable lifestyles*.”

Six articles are published in the final Research Topic in the following order:  Bryant and Dillard's article on “The Impact of Framing on Acceptance of Cultured Meat” highlights impressively how the perception of any novel food-related concept depends upon the way and context it is being presented. By using three different frames on cultured meat, i.e., “societal benefits,” “high tech,” and “same meat” they illustrate how the overall reception of these novel materials is context dependent.

The work by Bodiou et al. from the laboratory of Mark Post and Mosa Meat BV explores technology development, discussing the role of “Microcarriers for Upscaling Cultured Meat Production.” Investigations into proper substrates and scaffolds for cultured meat are rapidly advancing. It is becoming clear, that any discussion about bioreactor design will have to be informed by the type of scaffolding and substrate that are being used to make biomass proliferate and differentiate. Later in the Research Topic, Bellani et al. return to this important point.

By choosing the title “Cultured Meat—*Are We Getting it Right*?” we aimed to encourage a critical assessment for this developing technology. In their review article “The Myth of Cultured Meat: A Review” Chriki and Hocquette provide exactly that, while keeping a balanced and analytical view on the topic.

“Sensorial and Nutritional Aspects of Cultured Meat in Comparison to Traditional Meat: Much to Be Inferred” by Fraeye et al. is a glimpse into the sensorial and organoleptic properties of future products to come. This paper also compares cultured meat to traditional meat from a tissue engineering and meat technological point of view.

A relevant question to emerge from this contribution might be: To what degree will cultivated meat have to mimic existing animal-based meat products such as cuts and steaks and ribs?

Bogueva and Marinova explore “Cultured Meat and Australia's Generation Z.” Interestingly, this contribution received a lot of attention—possibly because of the very local Australian aspects that are discussed in great detail. Concerns about masculinity and betraying Australia as a country of quality animal meat are raised. However, a significant number of young people (28%) are prepared to try cultured meat. Environmental and health concerns may encourage a broader section of society to embrace it as a novelty.

With their paper “Scale-Up Technologies for the Manufacture of Adherent Cells” Bellani et al. present their work on the overarching key issue in the cultured meat arena: scaling. Scaling at an affordable cost will be the key critical parameter to ensure the technology will prevail. The article gives a well-balanced overview of the different approaches in bioreactor design necessary to develop production plants for the upper kg range. Bioreactor design in the cultured meat field is becoming a formidable challenge for engineering departments across the globe.

The exploration and evolution of a second agricultural domestication (1) cannot be ignored any longer. It took mankind 10, 000 years to domesticate multicellular macroorganisms such as plants and animals. It will now potentially only take a few decades to domesticate the tissue analogues of these organisms at scale starting from the cellular level. The ambition to develop cultivated meat is only one facet in the growing domain of cellular agriculture and the discussion is about to reach a mainstream audience (2). As with many waves in the development of technologies, there are bumps along the way, there are dead-end roads, there are duplicate inventions and yet there is an underlying slow and steady way forward. It is disciplines such as biology and nutrition science that provide us with a deeper understanding of our physiological needs, states, and health requirements. Such understanding will eventually enable consumer acceptance of cellular agriculture. Overall, these advances are the result of the global necessity to renovate our approaches to nutrition and health systems toward providing for both individual and planetary health (3).

## Author Contributions

The author confirms being the sole contributor of this work and has approved it for publication.

## Conflict of Interest

The author declares that the research was conducted in the absence of any commercial or financial relationships that could be construed as a potential conflict of interest.
